# Brain BOLD MRI O_2_ and CO_2_ stress testing: implications for perioperative neurocognitive disorder following surgery

**DOI:** 10.1186/s13054-020-2800-3

**Published:** 2020-03-04

**Authors:** W. Alan C. Mutch, Renée El-Gabalawy, Lawrence Ryner, Josep Puig, Marco Essig, Kayla Kilborn, Kelsi Fidler, M. Ruth Graham

**Affiliations:** 1 Department of Anesthesiology, Perioperative and Pain Medicine, Max Rady College of Medicine, University of Manitoba, 2nd Floor, Harry Medovy House, 671 William Ave., Winnipeg, MB R3E 0Z2 Canada; 2 Canada North Concussion Network, http://www.CNCN.ca; 3 Department of Clinical Health Psychology, University of Manitoba, Winnipeg, Canada; 4 Department of Radiology, University of Manitoba, Winnipeg, Canada; 5 Department of Physics, University of Manitoba, Winnipeg, Canada

**Keywords:** Anesthesia, CO_2_, O_2_, BOLD, MRI, Cognitive dysfunction, Critical care, Delirium, Mechanical ventilation

## Abstract

**Background:**

Mechanical ventilation to alter and improve respiratory gases is a fundamental feature of critical care and intraoperative anesthesia management. The range of inspired O_2_ and expired CO_2_ during patient management can significantly deviate from values in the healthy awake state. It has long been appreciated that hyperoxia can have deleterious effects on organs, especially the lung and retina. Recent work shows intraoperative end-tidal (ET) CO_2_ management influences the incidence of perioperative neurocognitive disorder (POND). The interaction of O_2_ and CO_2_ on cerebral blood flow (CBF) and oxygenation with alterations common in the critical care and operating room environments has not been well studied.

**Methods:**

We examine the effects of controlled alterations in both ET O_2_ and CO_2_ on cerebral blood flow (CBF) in awake adults using blood oxygenation level-dependent (BOLD) and pseudo-continuous arterial spin labeling (pCASL) MRI. Twelve healthy adults had BOLD and CBF responses measured to alterations in ET CO_2_ and O_2_ in various combinations commonly observed during anesthesia.

**Results:**

Dynamic alterations in regional BOLD and CBF were seen in all subjects with expected and inverse brain voxel responses to both stimuli. These effects were incremental and rapid (within seconds). The most dramatic effects were seen with combined hyperoxia and hypocapnia. Inverse responses increased with age suggesting greater risk.

**Conclusions:**

Human CBF responds dramatically to alterations in ET gas tensions commonly seen during anesthesia and in critical care. Such alterations may contribute to delirium following surgery and under certain circumstances in the critical care environment.

**Trial registration:**

ClincialTrials.gov NCT02126215 for some components of the study. First registered April 29, 2014.

## Background

Mechanical ventilation is a mainstay of management of patients in the operating room and critical care environment. Once implemented, the clinician may alter inspired O_2_ by fivefold and markedly modify end-tidal (ET) CO_2_ from the range seen in healthy subjects breathing spontaneously. The use of 100% oxygen during anesthesia induction and emergence and maintenance of anesthesia with inspired O_2_ concentrations of 50% are typical. Periods of both hypocapnia during mechanical ventilation and hypercapnia during the reestablishment of spontaneous ventilation are common.

We have previously hypothesized that alterations in ET CO_2_ common during anesthesia could contribute to perioperative neurocognitive disorder (POND) [1] in vulnerable individuals based on observed alterations in blood oxygenation level-dependent (BOLD) magnetic resonance imaging (MRI) during controlled CO_2_ “stress testing” [2–4]. Stress testing refers to an experimental procedure that exposes an individual to controlled alterations of respiratory gases in the awake state using a computer-controlled gas blender. Follow-up work confirms intraoperative hypocapnia as a marker for POND [5]. These findings were recently substantiated in a study where anesthesia was stabilized with multiple closed-loop controllers, with one controller limiting swings in ET CO_2_ thereby limiting hypocapnia during mechanical ventilation [6]. Arterial oxygenation was not considered as an independent factor in these studies, although it is well established that excessive inspired O_2_ can damage end organs [7–10]. Recently, intraoperative hyperoxia has been shown to increase the incidence of postoperative delirium in cardiac surgery patients [11]. Thus, alterations in both respiratory ET gases have been implicated in POND. Mechanisms of cerebral dysfunction from these ET gas changes remain unclear. Importantly, the neurophysiological interactions between commonly observed combined alterations in O_2_ and CO_2_ ET gas tensions that occur during intraoperative mechanical ventilation have not been well delineated.

We postulate that these alterations in respiratory gases can have significant effects on cerebral blood flow (CBF) and cerebral tissue oxygenation and may be implicated in cognitive dysfunction, including POND [1]. POND encompasses pre-existing problems such as mild cognitive impairment or dementia, acute postoperative delirium, and postoperative cognitive dysfunction, with the potential for learning and memory deficits in young children and a progressive downward arc in cognitive abilities in older adults after surgery.

In the present study, using blood oxygenation level-dependent (BOLD) and pseudo-continuous arterial spin labeling (pCASL) MRI, we examine the effects of controlled alterations in ET O_2_ and CO_2_ in various combinations commonly observed during anesthesia and surgery on cerebral oxygenation and CBF in healthy awake adults. Implications of these observed changes are discussed in relation to POND to facilitate future research in this developing area.

## Materials and methods

These studies relate to ongoing examination of volunteer subjects and patients undergoing surgical intervention and concussion management at the University of Manitoba (U of M), Winnipeg, Canada. The protocol was approved by the Biomedical Research Ethics Board (BREB), at the U of M, and a cohort of the study group was registered at ClincialTrials.gov for some components of the study—NCT02126215. For the present study, 12 healthy adult volunteers gave witnessed informed consent and underwent MRI while ET gases were manipulated in a controlled fashion with a RespirAct (a computer-controlled model-based prospective end-tidal gas mixer) [12, 13]. The controlled alterations in ET gases were selected to be a reflection of changes in respiratory gases commonly seen in the operating room. The volunteers had no neurological health issues nor were receiving psychotropic drugs for any psychiatric conditions. None had a history of POND with any previous surgery, nor had admission to an ICU requiring mechanical ventilation.

### End-tidal gas protocols

**Fig. 1:**
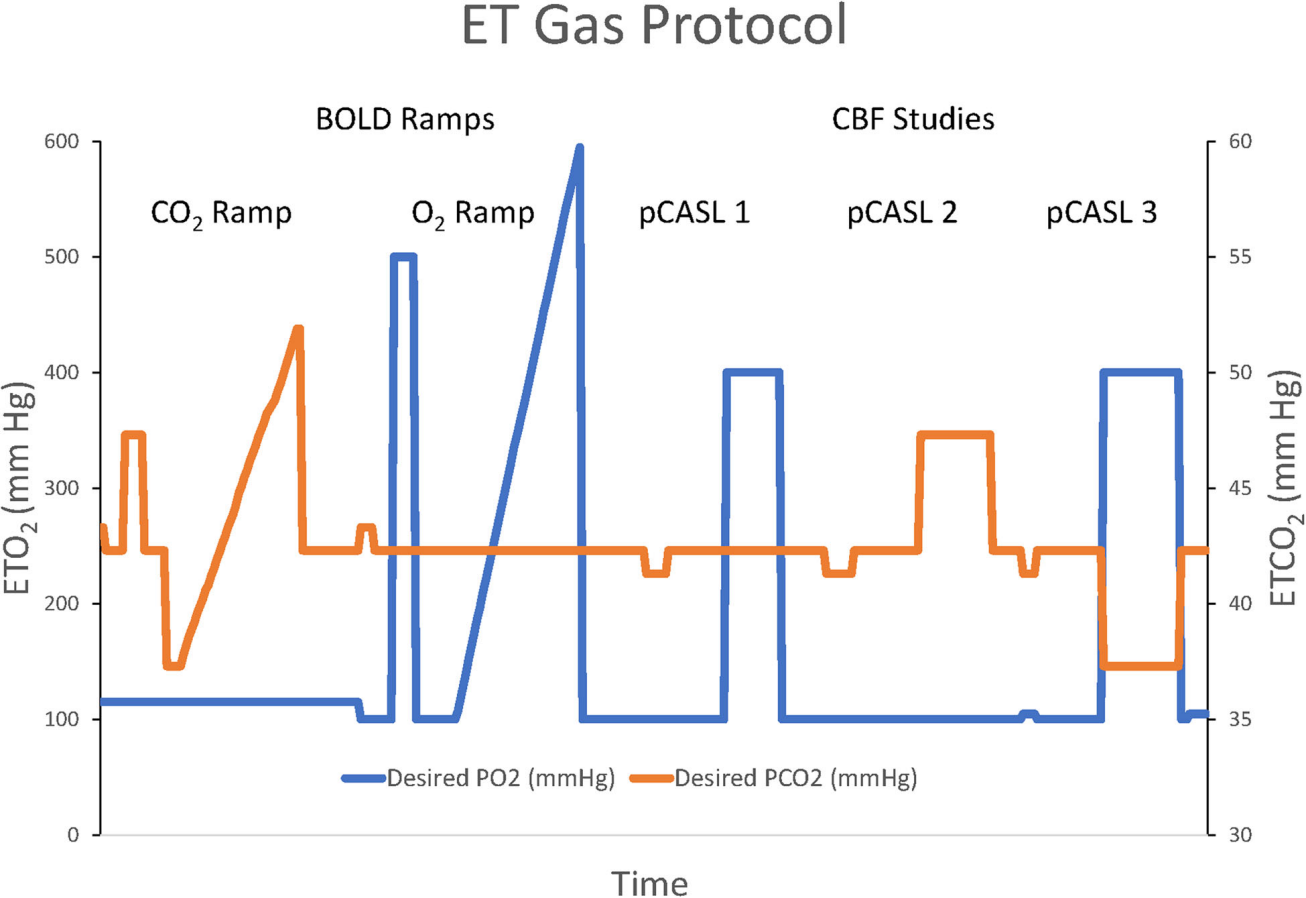
Display of the end-tidal gas control as described in the “Materials and methods” section for one subject. These alterations in gas tensions were deliberately chosen to be a reflection of those occurring during a typical anesthetic

Following a brief overview, subjects had an air-tight plastic mask affixed to their face. They then were positioned on the imaging table, and a prep sequence was run on the RespirAct to determine the individual’s resting ET gases, breathing frequency, and tidal volume. Once determined, the subject was positioned in the magnet bore. Figure 1 illustrates the time sequence of ET gas manipulations during imaging. BOLD images were acquired at baseline ETCO_2_ and during a sequence of square wave and ramp changes in ETCO_2_ from 5 mmHg below to 10 mmHg above baseline with O_2_ “clamped” or constant at 100 mmHg, followed by a sequence of square wave and ramp increases in O_2_ to maximum value (shown as 600 mmHg in Fig. 1) at baseline ETCO_2_. Then, pCASL images were acquired at baseline and during a square wave increase in ETO_2_ to 400 mmHg with ETCO_2_ at baseline, 5 mmHg square wave increase in ETCO_2_ with ETO_2_ clamped at 100 mmHg and finally during a 5-mmHg square wave decrease in ETCO_2_ with ETO_2_ at 400 mmHg. Baseline ETCO_2_ and O_2_ were restored between each sequence. The CO_2_ and O_2_ ramp sequences during BOLD imaging are easily tolerated and give a smooth second-by-second assessment of alterations in brain oxygenation over ET gas changes commonly seen during intraoperative mechanical ventilation. The pCASL sequences permit direct examination of the changes in CBF (the flow delta effect) that occur with the alterations in ET gases as described in the three pCASL sequences listed above—alterations in ET gases routinely seen during anesthetic management. Using the imaging protocol chosen, we can observe the dynamic effects on brain oxygenation during rigidly controlled increases in each of the two ET gases while the other ET gas is held at a constant tension through BOLD imaging and the alterations in true CBF with such changes by examination of the CBF delta with the pCASL imaging. A more comprehensive analysis of the relationship between BOLD and pCASL imaging is given in Supplemental File 1, and evidence offered that BOLD imaging is a robust proxy for CBF for the ET gas changes studied here.

### Imaging protocols

Images were acquired using a Siemens Verio 3.0 T MR scanner with a 12-channel phased-array head coil. Anatomical imaging was done without manipulation of ET gases using a sagittal 3D T1 magnetization-prepared rapid acquisition with gradient echo (MPRAGE) (whole brain coverage, matrix 256 × 256, slice thickness 2.2 mm, no interslice gap, voxel size 2 mm × 2 mm × 2 mm). Axial gradient recalled echo planar (GRE) sequences were obtained to screen for cerebral microhemorrhages as well as gradient field mapping. Cerebrovascular reactivity (CVR) to alterations in ET CO_2_ and O_2_ was assessed using continuous BOLD MRI during the complete period of MPET CO_2_ and O_2_ targeting (the full 11-min sequence or 660 s—see below for fuller description). BOLD MRI data were acquired using a T2*-weighted single-shot gradient echo pulse sequence with echoplanar readout (field of view 24 cm × 24 cm, matrix 64 × 64, time to repeat (TR) 2000 ms, time of echo (TE) 30 ms, flip angle 85°, slice thickness 5.0 mm, interslice gap 2.0 mm, voxel size 3.75 × 3.75 × 6 mm, number of temporal frames = 330, 10 s of initial imaging data was discarded to allow for equilibration). The BOLD GRE images were obtained using Siemens proprietary software for prospective motion correction (PACE). Global mean resting CBF was assessed using pseudo-continuous arterial spin labeling (pCASL) that included an initial M0 scan—Siemens ep2d_pCASL—echo-planar readout (field of view 24 cm × 24 cm, TR 8000 ms, TE 12 ms, contrast with a flip angle 90°, 20 slices, CASL method—multislice, label offset 90 mm, post label delay 1200 ms, crusher gradient 0 s/mm2, and voxel size 3.8 mm × 3.8 mm × 5.0 mm). The formal pCASL sequence then followed consisting of an echo-planar readout (field of view 24 cm × 24 cm, TR 4000 ms, TE 12 ms, contrast with a flip angle 90°, 20 slices, slice thickness 5.0 mm, CASL method—multislice, label offset 90 mm, post-label delay 1200 ms, crusher gradient 0 s/mm^2^, voxel size 3.8 mm × 3.8 mm × 5.0 mm). Imaging duration was for 3 min. The first two labeled–non-labeled pairs were discarded. The pCASL sequence was repeated × 3 for cerebral blood flow determination during the various end-tidal gas protocols after re-equilibration of ET gases to baseline. Total imaging time was approximately 45–60 min/session.

### Post-processing of images

**Fig. 2:**
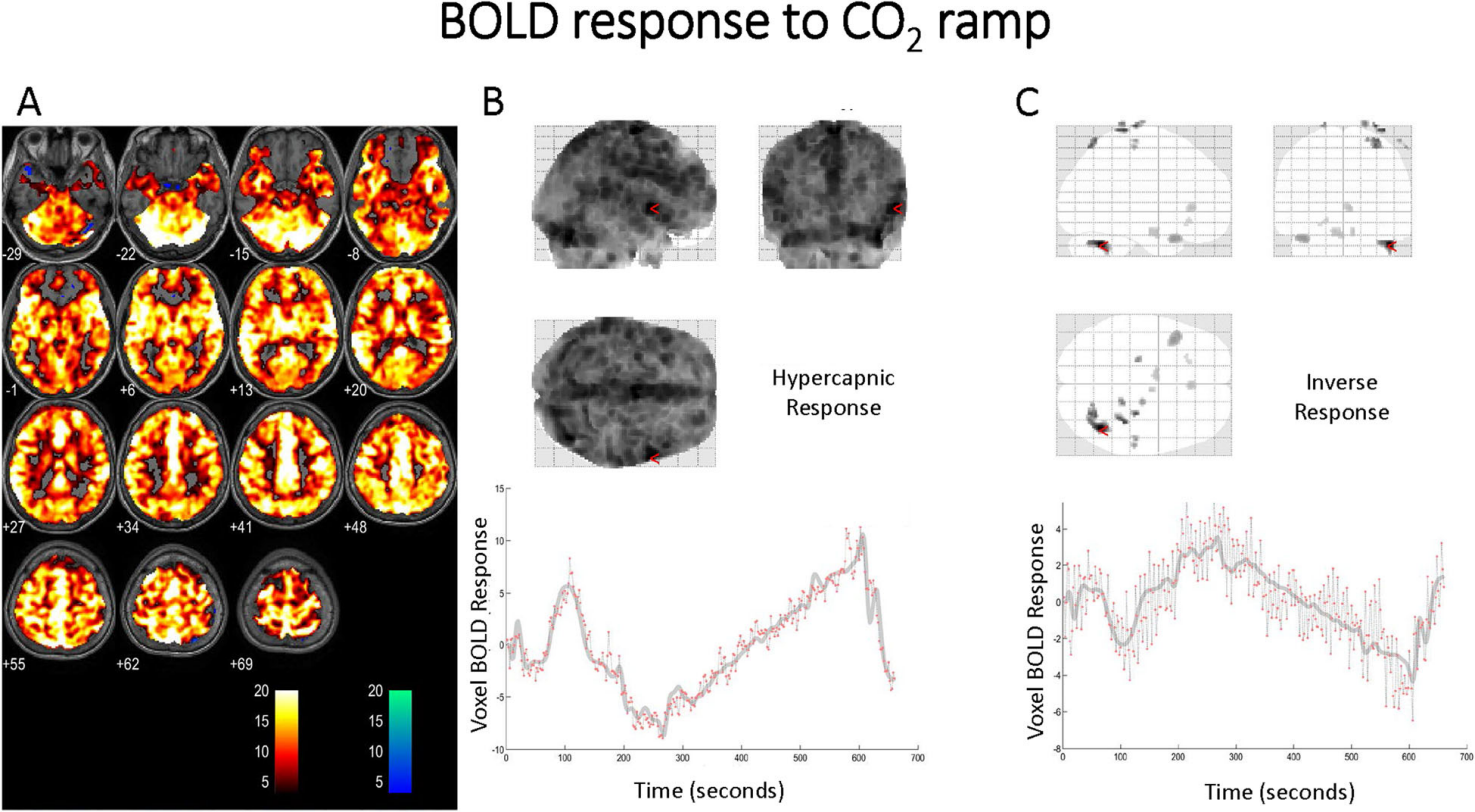
**a** BOLD response to the CO_2_ ramp with the ETO_2_ tension clamped at ~ 100 mmHg. The expected response with increased BOLD signal to the ramp stimulus is depicted by orange voxels. A diffuse response is seen. The scale is *t* scores based on fit to the GLM from SPM first-level analysis with “hotter” colors indicating a better fit to the GLM. The blue voxels depict the inverse response with “colder” colors indicating a better fit to the inverse response to the CO_2_ ramp. The *t* score had to exceed 3.11 (*p* = 0.001) to be colorized. **b** The response of one voxel to the CO_2_ ramp. The incremental and rapid increase with the step change in CO_2_ (gray scale) is seen by the red dots representing the BOLD scan signal intensity at that moment in time. **c** The inverse response of one voxel to the CO_2_ ramp. The incremental and rapid decrease with the step change in CO_2_ (gray scale) is seen by the red dots representing the BOLD scan signal intensity at that moment in time. These data are from subject 3

The images were converted to nifti (.nii) files, and using custom-written batch files preprocessing was accomplished using standard statistical parametric mapping version 8 (SPM8) pipelines. BOLD images were re-aligned, co-registered to the MPRAGE images, normalized, and smoothed with a 5 × 5 × 5-mm smoothing kernel. The BOLD image sequences at each measured voxel were then fit to a general linear model (GLM) based on regression fit to the 660-s ET CO_2_ and O_2_ ramps respectively. This was accomplished by interpolating the ET value to each of the 330 BOLD image signal outputs per voxel obtained during the imaging sequence. See Figs. 1 and 2 for an example of the curve fits for the BOLD signal output to the CO_2_ and O_2_ ramp outputs respectively. Both the expected increase in BOLD signal (labeled as hyper-responsiveness—for hypercapnia and hyperoxia responses) and the inverse responses (where the BOLD signal output was negative to the expected response) were examined at various *p* values and *t* scores. First- and second-level analyses were undertaken for the images; the approach used can be reviewed in the SPM8 manual—https://www.fil.ion.ucl.ac.uk/spm/doc/spm8_manual.pdf. Various masks were applied to the images to examine specific regions. The pCASL images were processed using the ASLtbx developed by Wang [14]. Cerebral blood flow was determined at the baseline settings for each of the three runs and the altered CBF determined for CVR assessment of the block-designed change in flow. Flow maps were determined for baseline settings, the block-designed stress, and the difference between. As per the customary SPM approach, second-level analysis was undertaken to assess mean responses for CBF. As a representative area associated with cognition and potential for dysfunction with POND, the hippocampus, both right and left, was selected as a region of interest (ROI) in the hyperoxic-hypocapnic flow sequences. The Montreal Neurologic Institute (MNI) mask for the hippocampi was determined from the Wake Forest Pick Atlas. Voxel counts and voxel output values were obtained by using the masks developed from the Pick Atlas and then collated with the use of the post-processing software MarsBaR (MARSeille Boîte À Région d’Intérêt).

## Results

Twelve studies were undertaken. In all, CO_2_ and O_2_ BOLD GRE ramp protocols were completed. In nine, pCASL measurements were obtained. In these, the first two pCASL protocols were completed in all cases, and all three pCASL protocols were completed in six subjects (these individuals were able to hyperventilate on demand to obtain the hypocapnic response). There were no complications noted following completion of the protocols.

Subject demographics are seen in Table 1. One subject had a history of migraine headaches, and one subject had a prior history (remote) of concussion.

**Table 1:** Subject demographics

Study number	Age	Sex	Status	Medications	Resting BP
1	42	M	Migraines	Nil	122/77
2	29	M	Remote concussion	Nil	118/69
3	61	F	Healthy	Antibiotics	135/83
4	45	M	Healthy	Nil	138/86
5	57	F	Healthy	Nil	141/89
6	53	F	Healthy	Nil	145/77
7	62	M	Healthy	Nil	155/84
8	44	M	Healthy	Nil	110/70
9	20	F	Healthy	Nil	110/66
10	54	F	Healthy	Nil	130/82
11	56	F	Healthy	Nil	138/74
12	52	F	Healthy	Nil	112/65
**Mean**	**48**				
**SD**	**13**				

The ET gas results are shown in Table 2. All subjects had ET gas control close to their planned alterations as depicted in Fig. 1. A plateau was seen for the upper limit of ET O_2_ for all with a maximum in the range of 475 mmHg in the O_2_ ramp protocol.

**Table 2:** End-tidal gas targeting

Study number	Baseline CO_2_	Hypocapnic CO_2_	Ramp peak CO_2_	Baseline O_2_	Ramp peak O_2_
1	45.5	41.2	53	103.5	534
2	40.2	35.5	50.1	110	411
3	37.8	31	46.6	102.5	489
4	38.1	32.3	45.2	99.7	461
5	43.2	38.3	53.1	101.8	427
6	41.7	36.6	51.9	100.1	461
7	40.9	37.6	53.1	110.2	415
8	48.1	43.4	57.4	99.8	409
9	40.8	36.9	49	104.2	463
10	42.9	37.1	53.8	101.9	522
11	41.6	38.6	51.2	101.5	541
12	43.1	38.9	52.1	99.5	565
**Mean**	**42.0**	**37.3**	**51.4**	**102.9**	**474.8**
**SD**	**2.9**	**3.4**	**3.3**	**3.7**	**55**

Figure 2 shows a representative BOLD response in a single subject to the CO_2_ ramp protocol. The corresponding first-level analysis for the CO_2_ ramp for all subjects is shown in Table 3. The hyper (anticipated) response represents those voxels manifesting an increase in cerebral oxygenation to the hypercapnic stimulus. An increase in BOLD signal (hyper-response) was present in over 87 ± 4% of the voxels imaged indicating that the expected increase in brain oxygenation due to CO_2_-induced vasodilation was robust. Voxels were considered reactive (hyper) to the CO_2_ stimulus if the fit to the general linear model (GLM) exceeded a *t* score of 3.11 (*p* = 0.001; for that voxel). A small number of voxels manifested an inverse response—a decrease in BOLD signal—to the hypercapnic stimulus and was present in 0.6 ± 0.7% of all voxels. Voxels were considered to manifest an inverse response if the fit to the GLM exceeded a *t* score of 3.11 (*p* = 0.001). The distribution of voxels manifesting this inverse response was largely located in the deep white matter. The *p* = 1.0 value is a means to tabulate all of the voxels imaged to obtain the percentage responses listed.

**Table 3:** First-level analysis CO_2_ ramp

Subject	Voxel counts		Total imaged	% response to stimulus	
	Hyper	Inverse	*p* = 1.0	Hyper	Inverse
1	171,535	664	188,356	91.1	0.4
2	176,211	1088	191,764	91.9	0.6
3	149,610	475	171,395	87.3	0.3
4	147,991	0	175,213	84.5	0.0
5	140,787	879	163,827	85.9	0.5
6	143,022	4556	180,009	79.5	2.5
7	135,339	118	165,881	81.6	0.1
8	136,464	466	162,218	84.1	0.3
9	162,694	1442	189,248	86.0	0.8
10	165,279	294	178,274	92.7	0.2
11	155,785	1225	175,290	88.9	0.7
12	147,928	1750	170,293	86.9	1.0
**Mean**	**152,720**	**1080**	**175,981**	**86.7**	**0.6**
**SD**	**13,586**	**1219**	**9970**	**4.0**	**0.7**

Total with *p* = 1.0 all voxels imaged/subject

*Hyper* expected hypercapnic GLM fit to CO_2_ ramp, *Inverse* inverse response GLM fit to CO_2_ ramp, *p* = 0.001, *Raw voxel counts* gray matter + white matter inclusive mask

**Fig. 3:**
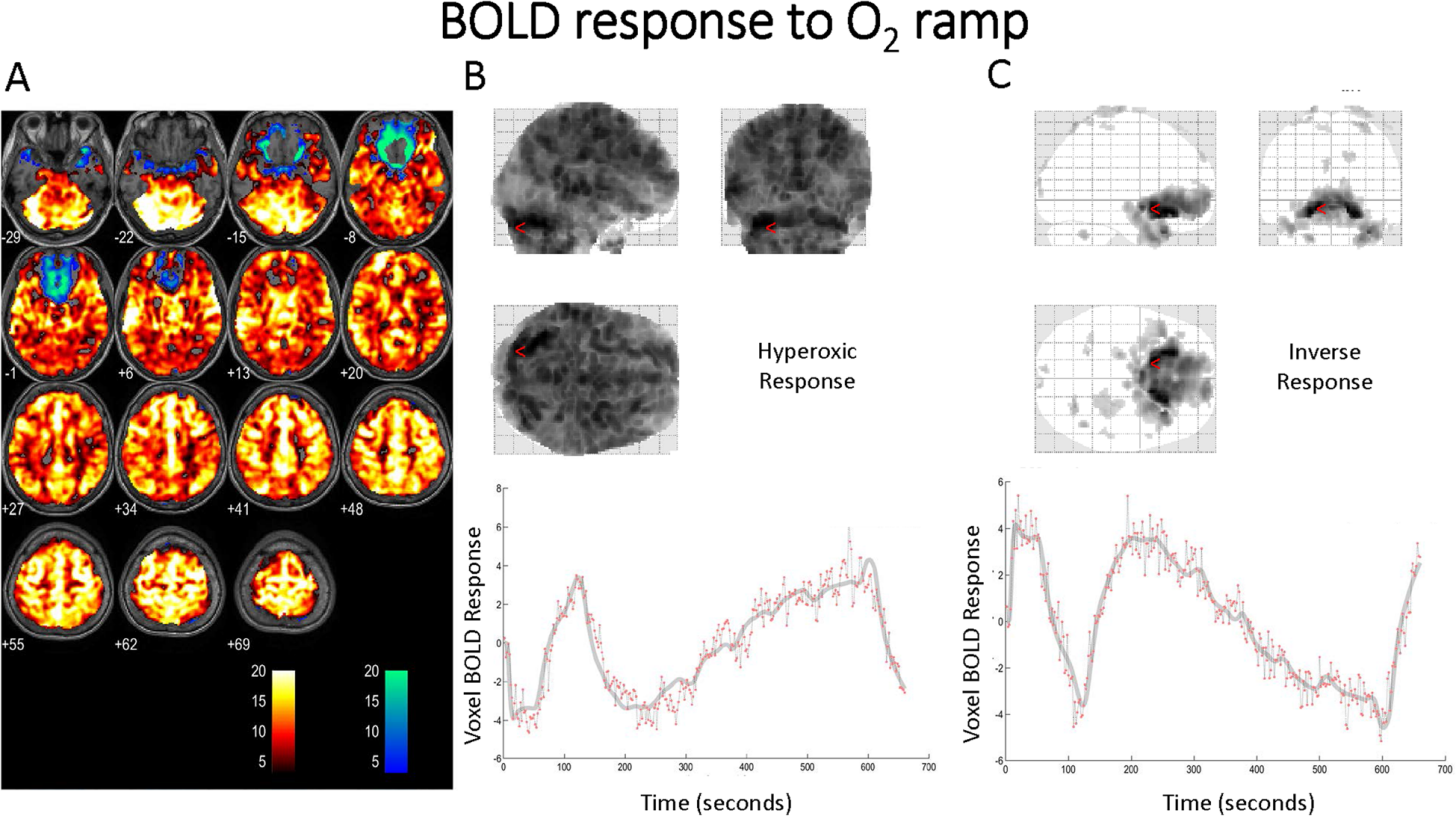
**a**–**c** BOLD response to the O_2_ ramp with the ETCO_2_ tension clamped at ~ 43 mmHg. The expected response to the ramp stimulus is depicted by orange voxels. A diffuse response is seen. The scale is *t* scores based on fit to the GLM from SPM first-level analysis. The blue voxels depict the inverse response. The *t* score had to exceed 3.11 (*p* = 0.001) to be colorized. **b** The response of one voxel to the O_2_ ramp. The incremental and rapid increase with the step change in O_2_ (gray scale) is seen by the red dots representing the BOLD scan signal intensity at that moment in time. **c** The inverse response of one voxel to the O_2_ ramp. The incremental and rapid decrease with the step change in O_2_ (gray scale) is seen by the red dots representing the BOLD scan signal intensity at that moment in time. These data are from subject 3

**Fig. 4:**
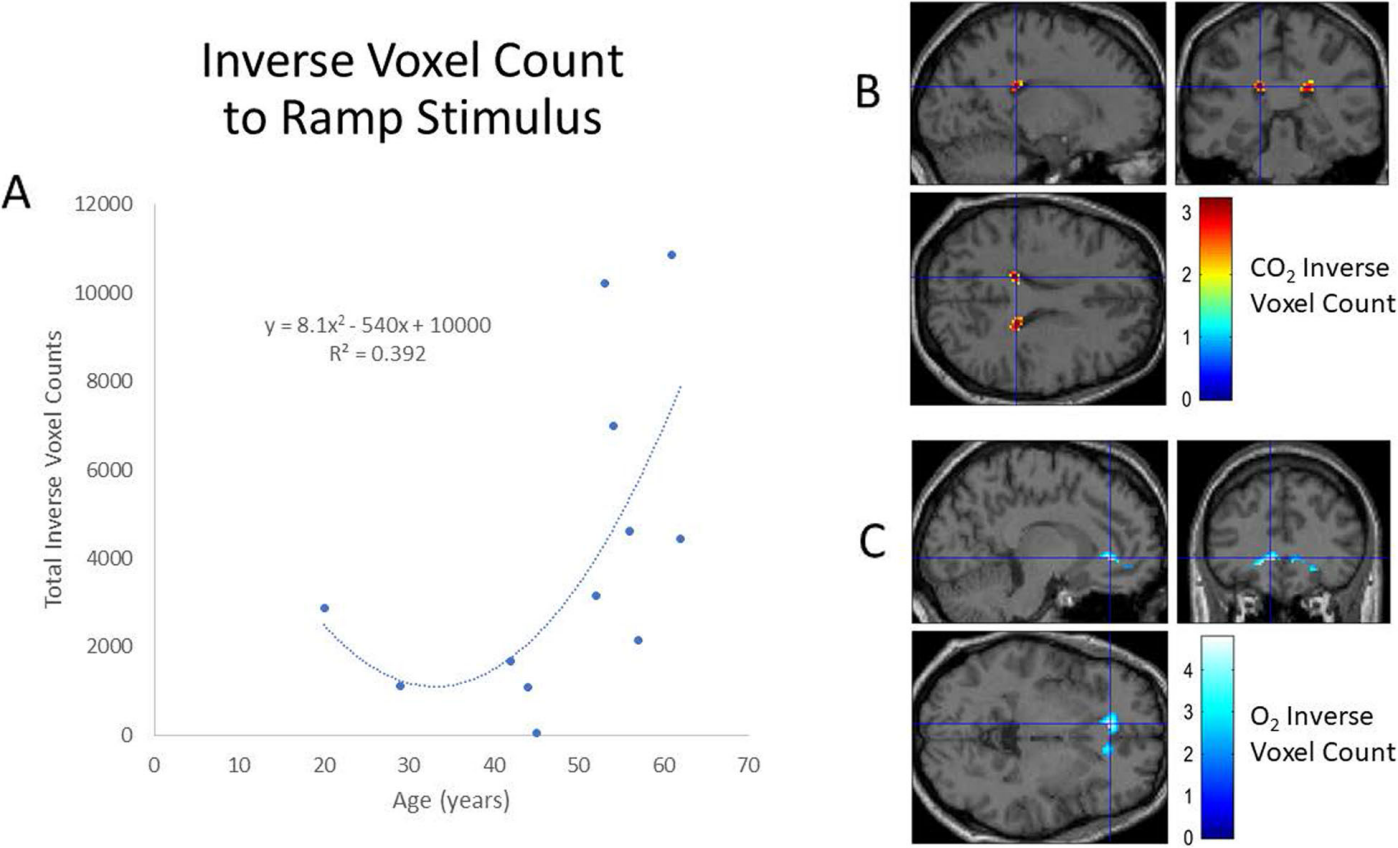
**a**–**c** In **a**, the relationship between inverse voxels to CO_2_ and O_2_ and age is shown. These data best fit a second-order polynomial; *p* = 0.029. **b** Second-level group analysis based on the inverse response to the CO_2_ ramp (orange voxels). The statistically significant voxels are in the deep periventricular white matter. **c** Second-level group analysis based on the inverse response to the O_2_ ramp (orange voxels). The statistically significant voxels are in the prefrontal and medial temporal regions

Figure 3 demonstrates a representative BOLD response in a single subject to the O_2_ ramp protocol. The corresponding first-level analysis for the O_2_ stimulus for all subjects is shown in Table 4. Similar to the pattern of response seen with CO_2_, a robust increase in BOLD signal is evident with 88 ± 9% of the voxels demonstrating the expected hyper-response. Again, as with the CO_2_ stimulus, a small number of voxels (1.7 ± 1.8% of total) demonstrated an inverse BOLD response to hyperoxia. The distribution of voxels with a decrease in BOLD signal during hyperoxia differed from those manifesting an inverse response to CO_2_ and was largely located in the deep prefrontal, frontal, insular, and hippocampal regions. See Fig. 4 for the respective distribution of the mean inverse CO_2_ and O_2_ response and a graphic representation of total inverse voxel response versus age. This correlation best fit a second-order polynomial; *r* = 0.664, *p* = 0.019.

**Table 4:** First-level analysis O_2_ ramp

	Voxel counts		Total imaged	% response to stimulus	
Subject	Hyper	Inverse	*p* = 1.0	Hyper	Inverse
1	167,536	1018	189,485	88.4	0.5
2	162,894	37	192,232	84.7	0.0
3	104,446	10,378	172,364	60.6	6.0
4	164,438	49	174,910	94.0	0.0
5	158,102	1280	165,354	95.6	0.8
6	161,567	5672	179,049	90.2	3.2
7	143,566	4326	166,975	86.0	2.6
8	146,776	618	162,406	90.4	0.4
9	175,849	1432	189,268	92.9	0.8
10	163,619	6699	179,612	91.1	3.7
11	160,986	3395	175,470	91.7	1.9
12	158,532	1409	170,872	92.8	0.8
**Mean**	**155,693**	**3026**	**176,500**	**88.2**	**1.7**
**SD**	**18,258**	**3194**	**9822**	**9.2**	**1.8**

Total with *p* = 1.0 all voxels imaged/subject

*Hyper* expected hyperoxic GLM fit to O_2_ ramp, *Inverse* inverse response GLM fit to O_2_ ramp *p* = 0.001, *Raw voxel counts* gray matter + white matter inclusive mask

**Fig. 5:**
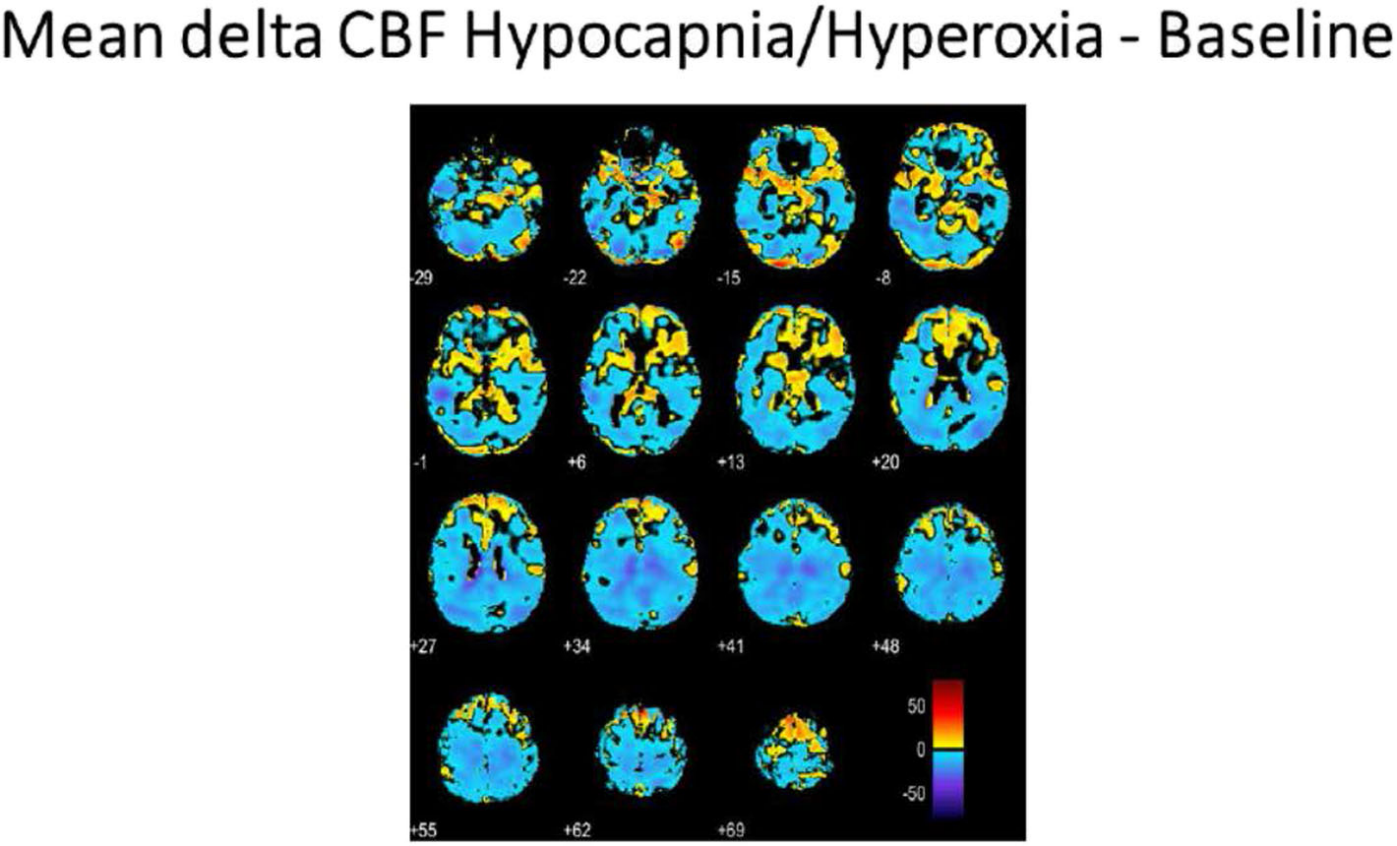
Mean delta CBF for hypocapnia/hyperoxia—baseline for the six subjects having this imaging sequence. A diffuse decrease in CBF is seen. This ET gas sequence is a common occurrence during anesthesia

**Fig. 6:**
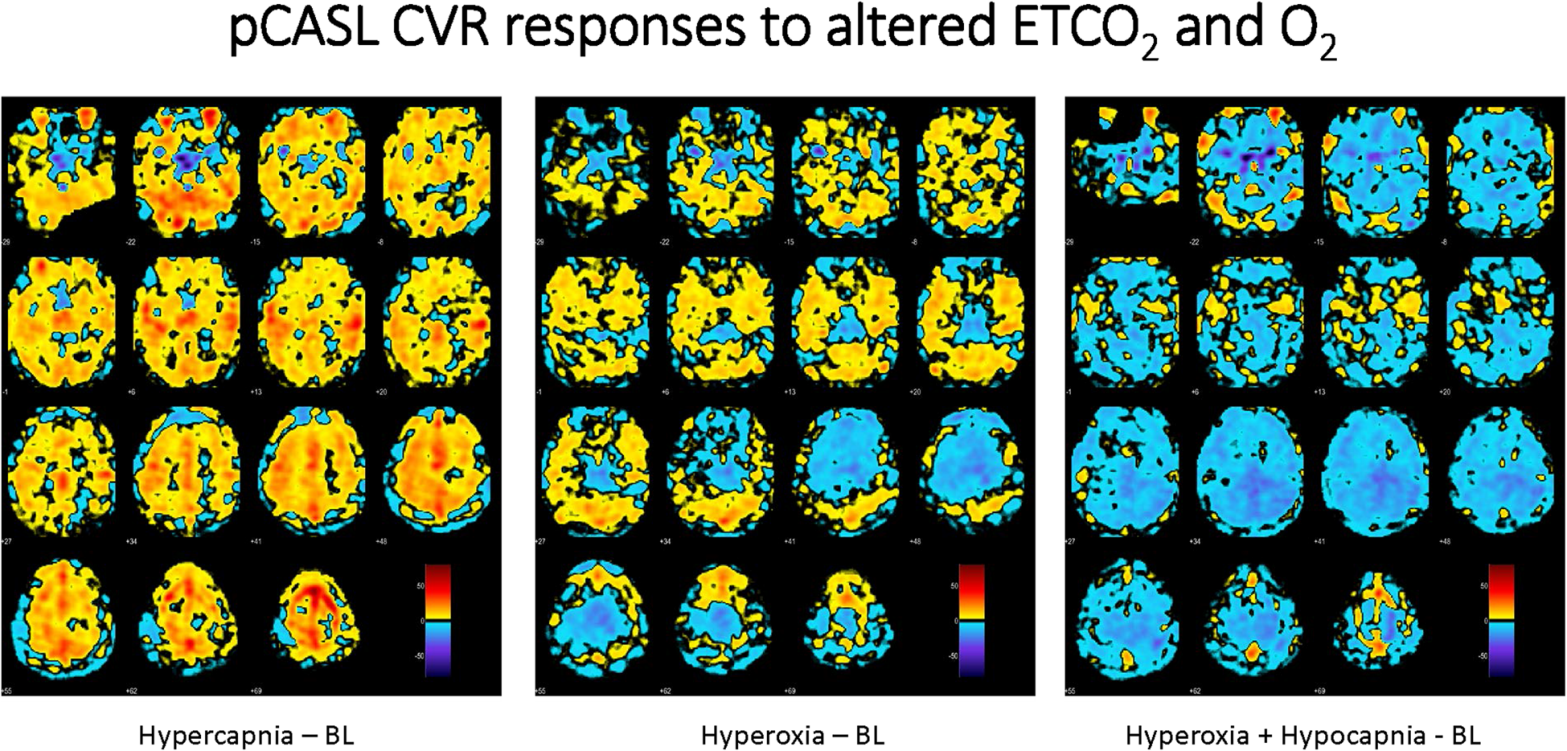
**a**–**c** Dynamism of the response of CBF to alterations in ET gases in a single subject. **a** CVR difference map of hypercapnia—baseline settings at normoxia. **b** CVR difference of hyperoxia—baseline settings at normocapnia. **c** CVR difference of hyperoxia/hypocapnia—baseline. The latter situation is often present during anesthesia. The large decrement in regional CBF is diffusely evident. These data are from subject 10

**Fig. 7:**
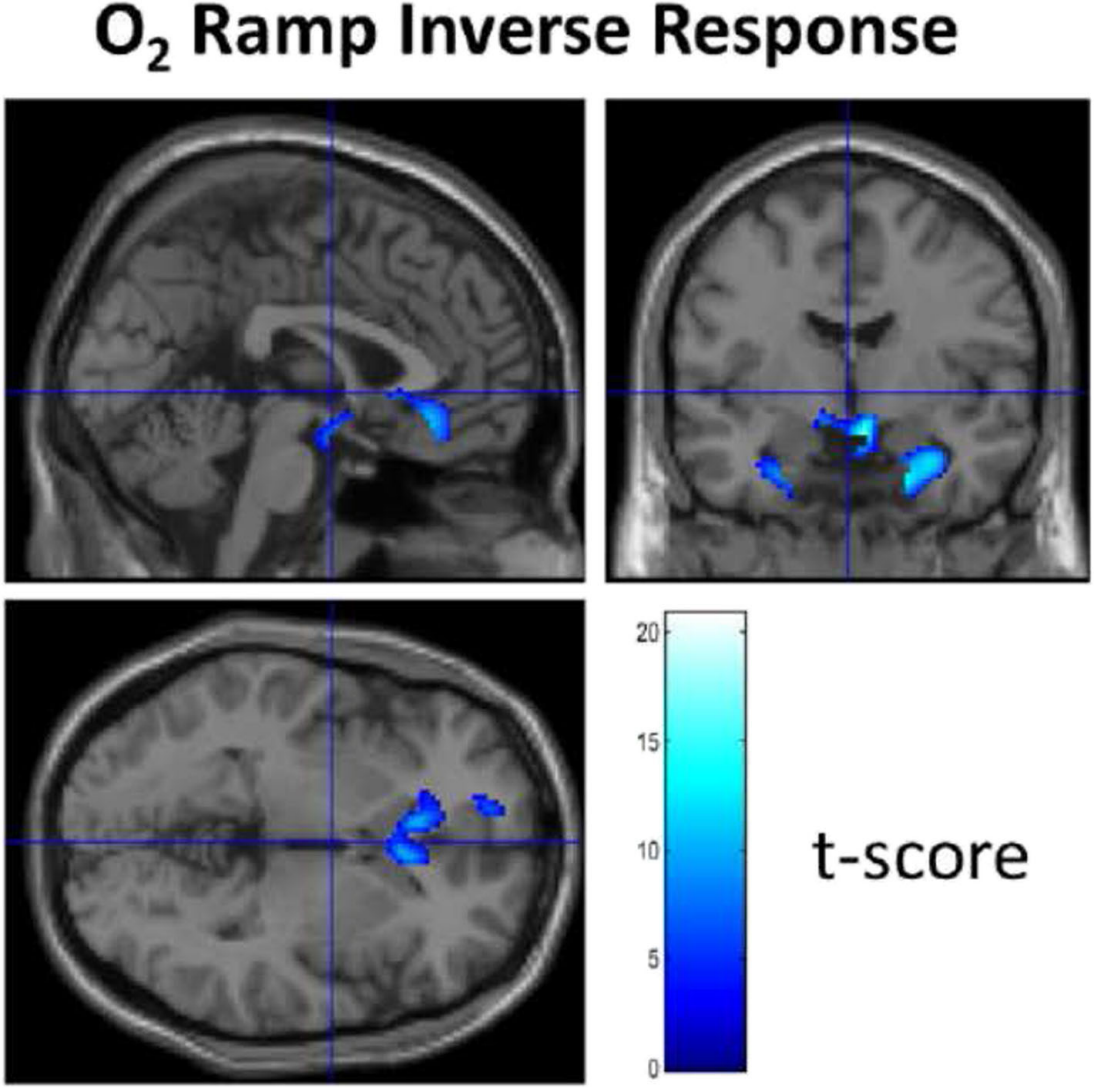
**a**–**c** A single subject study of the inverse response to the O_2_ ramp mapped on to their anatomic image in the sagittal, axial, and coronal views. Bilateral inverse response to the increase in O_2_ tension is seen in the hippocampi and midline in the hippocampal commissure. These data are from subject 10

The mean baseline gray matter flow was 42.8 ± 10.0 mL/100 g/min and white matter flow 15.5 ± 13.4 mL/100 g/min, and these values are similar to recently published results using similar methodology [15]. The group delta response to hypocapnia/hyperoxia as a combined stimulus versus baseline is shown in Fig. 5. An individual response to the pCASL protocol for the anesthesia-related alterations in ET gases is shown in Fig. 6. In this subject, a marked increase in CVR response is evident during hypercapnia at isoxia (A), a decrease in CVR response with hyperoxia at isocapnia (B) and a more marked decrease in CVR response when hyperoxia is combined with hypocapnia (C). An individual subject’s sagittal, axial, and coronal mapping of the distribution of inverse voxel response to the O_2_ ramp is shown in Fig 7. The coronal voxels are in the hippocampus bilaterally, and the midline voxels are in the location of the hippocampal commissure. The mean blood flow for the hippocampi was 42.3 ± 8.2 mL/100 g/min at baseline and reduced to 33.9 ± 9.3 mL/100 g/min with hypocapnia/hyperoxia (*n* = 6; *p* = 0.019 paired *t* test).

Supplemental File 1 has commentary on the relationship between BOLD and pCASL CVR for the ET delta ranges utilized in this study. The utility of BOLD imaging as a robust proxy for flow changes is discussed. Recent results by Dodd et al. [16] provide further endorsement of the approach in this study using similar BOLD and pCASL imaging techniques confirming prior concussion work from our research group [17]. The influence of blood pressure on the CO_2_ and O_2_ ET gas stimuli chosen is also highlighted here.

## Discussion

Results from this study indicate significant neurophysiological changes in cerebral oxygenation and CBF with respiratory end-tidal gas manipulations, used as a controlled proxy for the alterations in respiratory gases routinely seen perioperatively. Of particular concern is the fact that these alterations occurred in healthy adults during much briefer periods of altered gas tensions than would be typical in the clinical setting. Earlier work provides evidence for intraoperative hypocapnia as a factor in the development of POND in vulnerable individuals [5]. A pilot study suggests that individuals who manifest regional abnormalities with inverse responses to CO_2_ imaging may be at greater risk [3]. Intraoperative cerebral hyperoxia has been shown to increase the incidence of delirium following cardiac surgery [11]. In the present study, the findings of inverse responses to both hypercarbia and hyperoxia and the synergistic decrement in CBF seen with combined hypocapnia and hyperoxia suggest that alterations in both CO_2_ and O_2_ may have considerable import regarding the conduct of mechanical ventilation during anesthesia. The potential for large variation in CBF with routine alterations in ET gases during the conduct of a normal anesthetic can be deduced by examination of the images from this study. Extension of earlier findings regarding intraoperative hypocapnia raises concern that intraoperative O_2_ management might also be considered a potential modifiable factor in the development of POND.

Alterations in CBF to respiratory gases are based on responsiveness of the cerebral vasculature to arterial tensions of CO_2_ and O_2_. In this study, we have used ET measures of these gases as a surrogate for the arterial tensions. Ito et al. [18] have shown that with the MPET device (RespirAct) as used in this study, ETCO_2_ is a very accurate surrogate for arterial CO_2_ over a considerable range of inspired O_2_ tensions. Such an observation adds robustness to our findings.

A marked dynamism for alteration in CBF and oxygenation with respiratory gas manipulation is clear. Individual inverse responsiveness to CO_2_ and O_2_ stimuli was seen at a robust statistical level in all subjects. The inverse response to CO_2_ was especially evident in the deep white matter [19]. The inverse response to the O_2_ stimulus was especially pronounced in the prefrontal, frontal, and medial temporal cortices where executive function resides and in the hippocampus where memory resides (see Figs. 4, 5, 6, and 7) potentially placing these critical areas of the brain at risk with commonly seen alterations in respiratory gases during anesthesia. The absolute number of voxels manifesting an inverse response was small in these healthy subjects with an age range of 20 to 62 years. Inclusion of older individuals and/or patients with underlying medical conditions may be expected to result in a greater proportion of voxels with an inverse response [3].

Mechanistically, the inverse responses to changing ET tensions of CO_2_ and O_2_ appear to differ. The “intracranial steal” seen with hypercapnia is well described [20] and represents shunting of blood away from regional areas with abnormal cerebral vascular responses that fail to dilate to adjacent regions that vasodilate normally in response to hypercapnia. The regional inverse response seen with hyperoxia may be more mechanistically complicated. Excessive regional vasoconstriction seems evident. This view is supported by the pCASL imaging demonstrating regional decrements in CBF in response to the hyperoxic stimulus as seen in Fig. 6. Surrounding areas with less vasoconstriction would receive higher flow. The inverse BOLD response suggests vasoconstriction of an intensity that venular offloading of oxygen from hemoglobin may be excessive, resulting in increased paramagnetic signal [21, 22]. This explanation suggests excessive regional vascular tone in these feeding vessels or a combination of vasoconstriction and neuronal activation with attenuated luxury perfusion as discussed below. It has been noted that frontal neurons in Alzheimer transgenic mice are vulnerable to hyperoxia through synaptic dysfunction and brain oxidative stress [23]. Vasoconstriction with hyperoxia is a proposed mechanism in this model [24, 25]. Regionally similar activated areas with hyperoxia have been shown in a pediatric cohort by Macey et al. [26]. They show both areas of increased and decreased fMRI signal with hyperoxia similar in distribution to those seen in our inverse responsiveness maps in Fig. 4. Macey et al. suggest that negative activation of these regions may be related to the sympathetic stimulation elicited by hyperoxia. The addition of 5% CO_2_ to the respiratory gas mixture resulted in a marked attenuation of the observed response, mechanistically associated with an increase in CBF and thereby greater BOLD signal intensity regionally, effectively countering the negative activation. In their study, the zero-meaned global time series signal with hyperoxia was detrended from each voxel’s signal [27]. In this manner, the respiratory gas stimulated signal was removed. As we were specifically interested in these signals to assess cerebral response to controlled changes in ventilatory ET gases commonly seen in the operating room and critical care environments, we have examined the effects not detrended, which accounts for the differences in results. Earlier work by Prisman and colleagues [28] has shown similar CVR maps in response to hyperoxia by altering O_2_ tension at stable CO_2_. Inverse responsiveness follows a similar distribution to what we have demonstrated. This work also indicates that the CVR to each millimeter of mercury change in O_2_ is approximately 1/60^th^ of that to CO_2_. While this is a small effect on a millimeter of mercury by millimeter of mercury basis when comparing the two gases, the O_2_ delta in the ramp protocol in our study is approximately 25-fold greater than the CO_2_ delta suggesting a total CVR effect near 40% of that seen with CO_2_ when comparing these CVR maps. As we show, it is the combined influence of hyperoxia and hypocapnia (discussed below), which is potentially of most concern clinically.

In a study by Lopez et al. [11], the presence of cerebral hyperoxia during cardiac surgery was associated with increased delirium. The authors measured various bio-markers indicative of oxygen free radical production but indicated that other mechanisms of damage through hyperoxia were likely. Our study indicates other mechanisms with hyperoxia and in addition indicates a deleterious synergism between hyperoxia and hypocapnia on CBF.

Particularly germane to critical care management of ET respiratory gases and cerebral outcomes is the recent ICU-ROX study [29]. In this study of conservative versus usual oxygen therapy based on lower planned pulse oximetry targets in the conservative group, a sub-group analysis indicated improved results in patients with hypoxic-ischemic encephalopathy when in the conservative group. Relative risk of death was 0.73 compared to the usual more liberal oxygen therapy, and unfavorable outcome on the Extended Glasgow Outcome Scale had a relative risk of 0.81. A recent editorial in JAMA highlights these and other findings and cautions that inappropriately titrated O_2_ can contribute to iatrogenic adverse effects [30]. The “appropriate use and dose of oxygen in acute care medicine” is becoming better defined.

Examination of the inverse BOLD responses seen in the BOLD signal curve fits in Figs. 2 and 3 indicates that the response to both CO_2_ and O_2_ alterations are rapid and incremental (essentially altering with each 2-s scan interval), indicating a very dynamic process. The pCASL findings indicate that regional vasoconstriction is marked with the combination of hyperoxia and hypocapnia (Figs. 5 and 6). As noted above, this combination of ET gas conditions is ubiquitous during the conduct of anesthesia. The curve fit examining the magnitude of inverse voxels fits a second-order polynomial correlated to increasing age although the sample size is small. The best fit to a paraboloid, however, is an interesting finding as the incidence of POND is bimodal at the extremes of life. Whether inverse voxel signatures are more evident in neonates and young children is an open question, although the work by Macey et al. described above indicates that similar conditions on exposure to hyperoxia in the presence of hypocapnia exist in children.

These control studies have been conducted in awake individuals, so the impact of anesthesia—from both volatile agents (known cerebral vasodilators) and intravenous agents (known cerebral vasoconstrictors with the exception of ketamine)—may alter the response to CO_2_ and O_2_ as seen here. However, a carefully done study in rats anesthetized with isoflurane (a volatile agent) reports many of the same BOLD and CBF findings using similar imaging approaches, suggesting that the respiratory gas effects on cerebral perfusion translate to the anesthetized state with volatile agents [31]. Recent work by Venkatraghavan et al. [32] shows marked fluctuations in brain BOLD and intracranial steal in four patients undergoing propofol anesthesia using a similar CO_2_ ramp protocol as in this study, indicating that the CO_2_ responsiveness we describe in the awake state persists under intravenous sedation/anesthesia.

We have previously demonstrated that intraoperative hypocapnia is associated with an increased incidence of POND [3, 5] and have proposed a stress-diathesis model to account for the finding. In this model, patients with an underlying neurocognitive propensity or diathesis, which may include an abnormal BOLD response to CO_2_, coupled with operative stress—intraoperative hypocapnia as one variable—are more likely to develop POND. ET O_2_ fluctuations were not considered as a potential factor in either study. The results of the present work suggest both O_2_ and CO_2_ changes may be relevant and have a synergistic interplay. MacDonald et al. [33] reported variations in CBF of nearly 100% in 10 healthy subjects exposed to hyperoxia. The heterogeneity of regional responses to hyperoxia suggests that one CBF does not fit all regions, either in gray or white matter. Thus, each individual may have a unique CBF response and regional heterogeneity as the ET gases are manipulated during surgery and potentially under any circumstance where mechanical ventilation is required. The heterogeneity of the inverse response from examination of Tables 3 and 4 corroborates the individual variability in response. This highlights the utility of such an analysis as group means may well mask individual susceptibility in relation to the stress-diathesis model we have proposed [3]. This may translate to differential risk for cognitive dysfunction based on a combination of premorbid factors and CBF responsiveness as outlined. Other human studies have indicated that hyperoxia induces cerebral vasoconstriction as a consequence of altered regional nitric oxide potentially contributing to the stress diathesis [34].

The identification of regional “intracranial steal” or “blue brain” on the BOLD images with a CO_2_ ramp protocol is increasingly being used to establish the need for and measure results from revascularization with superficial temporal artery to middle cerebral artery (STA-MCA) anastomoses in patients with severe intracranial vascular compromise [35, 36]. When successfully revascularized, such patients see improvements in CVR and significant resolution of the “blue brain” signal that indicated the area of regional steal. Thus, these abnormal regions as identified in our study have clear pathological correlates. It is also of interest that the inverse CO_2_ response can be demonstrated in healthy individuals but becomes more manifest with cerebral pathology [19]. There are currently no comparable studies regarding O_2_ ramp protocols, and the “blue brain” seen in this study with this imaging protocol but analogies to the findings with the CO_2_ ramp protocol are envisioned. Also, importantly not addressed here are the potential findings of an O_2_ ramp protocol in the presence of a clamped or constant period of hypocapnia. It is this sequence that is especially interesting as this respiratory gas profile is common during anesthesia and seen in the ICU. The short hypocapnic periods obtained in this study were obtained by having the subject spontaneously hyperventilating on command. An 11-min ramp sequence with stable hypocapnia by spontaneous hyperventilation for such a duration is not feasible in awake subjects. That said, our third pCASL sequence in six subjects where a 3-min period of hyperoxia with hypocapnia indicates a synergic diffuse decrease in CBF, which potentially could have deleterious effects in patients at risk of POND.

Despite these important findings, this study must be considered in light of a few limitations. As indicated, the sample was limited in size and age range and future research should aim to understand these dynamic processes in particularly vulnerable age groups at the extremes of life. Second, although this study has significant implications for POND, CBF alterations from CO_2_ and O_2_ changes on POND were not directly assessed. Third, as indicated, it is possible that other anesthetic agents and physiological factors may impact CBF intraoperatively, which were not evaluated in the current study. Fourth, although ET CO_2_ has been shown to be very close to the subject’s arterial CO_2_, minor differences may exist in values. Finally, although we focused on the hippocampus as an area of particular interest because of its role in cognition, other areas are implicated and should be investigated in future research.

## Conclusions

Based on these initial observations, we advance the hypothesis that ET respiratory gas control is an important management consideration during anesthesia. These concerns may be extended to mechanical ventilation in the critical care setting. Older patients appear to be at greater risk of “intracranial steal” of regional CBF with alterations in CO_2_ and O_2_, which corresponds with greater rates of POND in this population. But additional study, with patients older than those recruited here, is required to substantiate these results. Our previous study confirms an association between CO_2_ management and POND recently substantiated [3, 5, 6]. The imaging in the present study suggests that a combination of hyperoxia and hypocapnia may be particularly worrisome. We suggest that anesthetic and potentially critical care management may be optimized for cerebral health by attempting to maintain respiratory gases at or near the patient baseline values to stabilize CBF. An exception may occur during neurosurgical procedures and neuro-critical care where deliberate alterations to lower ET CO_2_ may be required. The marked CBF fluctuations as seen with controlled ET gas manipulation in this study imply that such CBF changes can occur under anesthesia or with sedation in an ICU setting where similar changes in ET gases routinely occur. Such large alterations in CBF and oxygenation may be a contributing factor to the development of POND in susceptible individuals. A clinical trial comparing rigorous maintenance of ET gases at a patient’s own baseline to standard anesthetic management to determine if the changes in CBF and oxygenation as seen in awake subjects in this study have an influence on the development of POND could be considered. Similar consideration could also be given to tighter control of end-tidal gases with mechanical ventilation in the critical care environment.

## Supplementary information

**Additional file 1: Supplemental File 1:** An analysis from earlier non-published work providing evidence that BOLD imaging is a very robust proxy for CBF changes for the ET gas sequences used in this study when directly comparing CVR as assessed by both methodologies. The stability of blood pressure over the ET gas range studied is also discussed here.

## Data Availability

The datasets used or analyzed during the current study are available from the corresponding author on reasonable request.
