# Supplementary material for: Brain BOLD MRI O2 and CO2 stress testing: implications for perioperative neurocognitive disorder following surgery
Source: Crit Care. 2020 Mar 4;24:76. doi: 10.1186/s13054-020-2800-3 (PMC7057494; doi:10.1186/s13054-020-2800-3)
Supplement: Supplementary file 1 — Additional file 1: Supplemental File 1: An analysis from earlier non-published work providing evidence that BOLD imaging is a very robust proxy for CBF changes for the ET gas sequences used in this study when directly comparing CVR as assessed by both methodologies. The stability of blood pressure over the ET gas range studied is also discussed here. [file 13054_2020_2800_MOESM1_ESM.pdf]

Supplemental File 1

Establishing the Experimental Model

Blood Pressure Considerations – Inhalation of high inspired concentrations of CO_2 can have significant hyperdynamic effects with increases in blood pressure (BP) and heart rate. As CO_2 is a potent cerebrovasodilatory stimulus if blood pressure increases in tandem, then CBF can increase markedly when the cerebral vessels are vasodilated due to increased hydraulic pressure or further augment the BOLD signal if analyzed as a surrogate for CBF. In this study we recorded the subjects resting blood pressure by cuff sphygmomanometry. A single measurement of BP at peak CO_2 delta is not really indicative of the potential dynamic range of BP that may occur during the CO_2 stress test. We have examined this more comprehensively in a pilot study (results not published) but discussed here. In the high magnetic field present while conducting MRI studies, continuous BP can only be obtained effectively by invasive monitoring through insertion of an arterial cannula. For healthy volunteers this was deemed too invasive an intervention. We have examined the hemodynamic effect of a hyperdynamic CO_2 delta of 10 mm Hg in a separate group of subjects with continuous cerebral oximetry, beat-to-beat blood pressure by finger plethysmography, time sequenced to a double step increase in CO_2 of 5 and then a further 5 mm Hg (the same magnitude of CO_2 delta as the total hypercapnic delta in the current publication). We conducted this study in 14 subjects. The relative increase in BP to the CO_2 stimulus was modest as highlighted in a representative figure below with the systolic and diastolic pressure changes seen with the alterations in end-tidal CO_2. The data stream was recorded at a fidelity of 20 hz. For the subjects studied the mean systolic BP rise from baseline to 10 mm Hg increase in CO_2 was 139±20 to 145±17 mm Hg respectively.

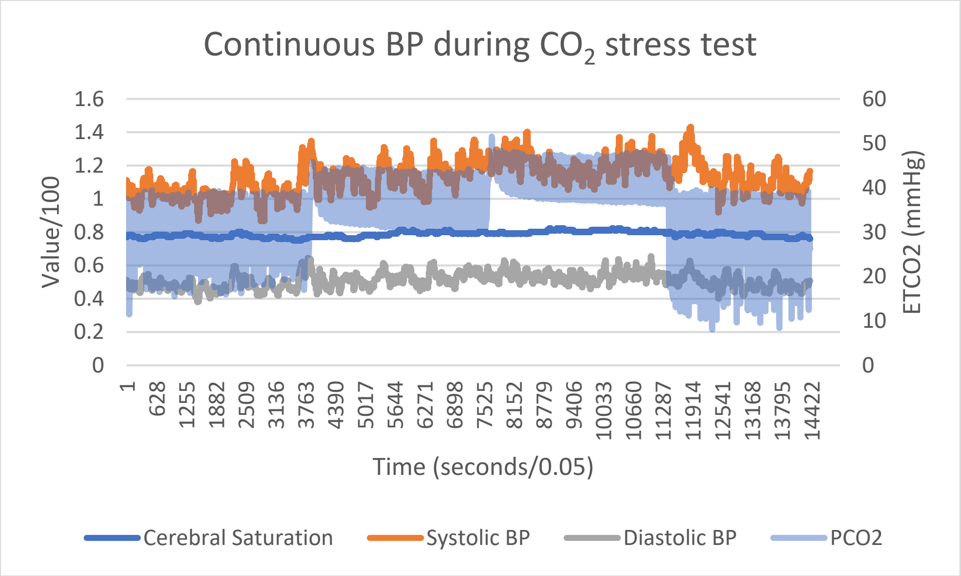

Figure 1

Consecutive imaging in 101 individuals (in 80 serial BP measurements during hypercapnic stimulus; controls and patients with concussion) revealed similar findings with modest changes in BP to the CO_2 delta as in this study. There is the occasional subject who responses vigorously to the CO_2 stimulus. Such

subjects can have increased BOLD responsiveness.[1] Such subjects could potentially be at greater risk of POND as discussed in this paper as having a greater diathesis risk to the CO_2 stress typical with anesthetic management. Such a subject is evident in Figure 6 of that manuscript. The CO_2 delta in that study was greater – 20 mm Hg. A more modest hemodynamic response in a second subject exposed to the same stimulus is shown in Figure 7 of that manuscript. Thus, in the current study the BP alterations with the hypercapnic stimulus of only 10 mm Hg appear modest and should represent a minor confounder on the data as interpreted.

BOLD imaging as a surrogate for CBF changes with altered end-tidal gases – The BOLD signal is a composite of a number of influences as principally measured at the venular level of the cerebral circulation. We have assumed that an increase or decrease in BOLD signal is a surrogate for alterations in CBF as a consequence of altered end-tidal gas tensions – especially so for CO_2. The BOLD signal can also be influences by changes in regional blood volume, alterations in hemoglobin and diffusion effects to identify some of the confounders. The working premise in this study is that the BOLD signal changes are due to altered CBF as $CMRO_2$ is minimally altered for the end-tidal gas tensions studied here. When establishing this research model we investigated the hypothesis that for the relative CO_2 delta under study the changes in BOLD signal were a robust surrogate for CBF changes. We examined the similarity in colorized maps of cerebrovascular reactivity to a 5 mm Hg step change in CO_2 for the BOLD signal and CBF as measured using the pCASL approach highlighted in this paper. Each of 15 adult subjects had a BOLD study followed after re-equilibration to baseline with a CBF study by pCASL to the same 5 mm Hg CO_2 stress test. The mean value voxel maps for the two approaches are shown below:

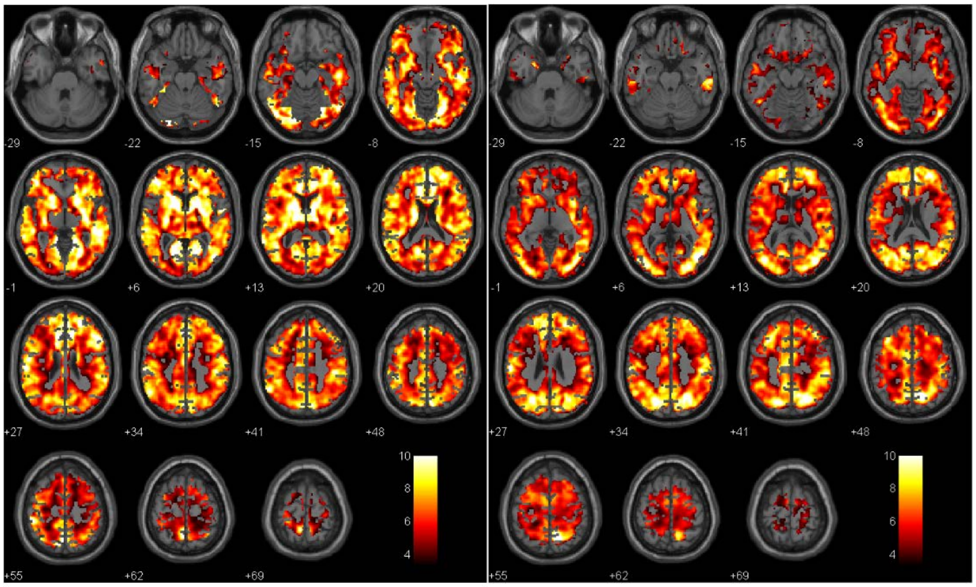

Figure 2 A and B

A: Raw BOLD CVR - response to a 5 mm Hg CO_2 stress test: n=15.

B: Raw pCASL CVR - response to a 5 mm Hg CO_2 stress test: n=15.

The mean group CVR responsiveness is very similar in distribution for the two imaging approaches, indicating that BOLD CO_2 responsiveness is a robust surrogate for CBF responsiveness. With BOLD imaging 83% of the voxels responded to the hypercapnic signal at the p=0.001 statistical level; with pCASL the response for activation was 43%. A greater response with BOLD imaging is expected as the

data series was analyzed over 180 scans for the 6-minute study period with 90 scans at each of the CO_2 end-tidal tensions and only 44 images were obtained for the pCASL sequence with only 22 images at the baseline and hypercapnic intervals. When the two approaches were compared directly at the 2nd level analysis only 4.1% of the BOLD voxels exceeded those as determined with pCASL imaging at the p=0.001 level. There were no voxels where the BOLD signal was statistically less than the pCASL voxels. These results would indicate that the BOLD response to CO_2 is a robust surrogate for CBF changes for the range of CO_2 delta as undertaken in this study.

References:

1. Mutch WAC, Mandell DM, Fisher JA, Mikulis DJ, Crawley AP, Pucci O, et al. Approaches to Brain Stress Testing: BOLD Magnetic Resonance Imaging with Computer-Controlled Delivery of Carbon Dioxide. PLoS One. 2012;7.
